# Spatial Frequency Maps in Human Visual Cortex: A Replication and Extension

**DOI:** 10.1101/2025.01.21.634150

**Published:** 2025-01-21

**Authors:** Jiyeong Ha, William F. Broderick, Kendrick Kay, Jonathan Winawer

**Affiliations:** 1 Department of Psychology and Center for Neural, New York University, NY, USA; 2 Flatiron Institute, Simons Foundation, NY, USA; 3 Center for Magnetic Resonance Research (CMRR), Department of Radiology, University of Minnesota, Minneapolis, MN, USA

## Abstract

In a step toward developing a model of human primary visual cortex, a recent study developed a model of spatial frequency tuning in V1 (Broderick, Simoncelli, & Winawer, 2022). The model is compact, using just 9 parameters to predict BOLD response amplitude for locations across all of V1 as function of stimulus orientation and spatial frequency. Here we replicated this analysis in a new dataset, the ‘nsdsynthetic’ supplement to the Natural Scenes Dataset (Allen et al., 2022), in order to assess generalization of model parameters. Furthermore, we extended the analyses to extrastriate maps V2 and V3. For each retinotopic map in each of the 8 NSD subjects, we fit the 9 parameter model. Despite many experimental differences between NSD and the original study, including stimulus size, experimental design, and MR field strength, there was good agreement in most model parameters. The dependence of preferred spatial frequency on eccentricity in V1 was similar between NSD and Broderick et al. Moreover, the effect of absolute stimulus orientation on spatial frequency maps was similar: higher preferred spatial frequency for horizontal and cardinal orientations compared to vertical and oblique orientations in both studies. The extension to extrastriate maps revealed that the biggest change in tuning between maps was in bandwidth: the bandwidth in spatial frequency tuning increased by 70% from V1 to V2 and 100% from V1 to V3, paralleling known increases in receptive field size. Together, the results show robust reproducibility of visual fMRI experiments, and bring us closer to a systematic characterization of spatial encoding in the human visual system.

## 1. Introduction

Many components of the visual system are selective to spatial scale, particularly in cortex. In both primates and cats, most V1 neurons exhibit relatively narrow bandpass tuning to spatial frequency (about 1 to 2 octaves), in contrast to the broader, low-pass tuning of LGN neurons (Campbell, Cooper, & Enroth-Cugell, 1969; De Valois, Albrecht, & Thorell, 1982; Foster, Gaska, Nagler, & Pollen, 1985). Preferred spatial frequency varies across neurons in V1, even within a small patch, indicating a multiscale visual representation (Campbell & Robson, 1968; De Valois et al., 1982). The distribution of preferred frequencies also varies across locations: for example, when averaged across local populations, peak spatial frequency decreases with eccentricity (De Valois et al., 1982; Issa, Trepel, & Stryker, 2000; J. A. Movshon, Thompson, & Tolhurst, 1978; Tootell, Silverman, Hamilton, Switkes, & De Valois, 1988; Xu, Anderson, & Casagrande, 2007). Spatial frequency tuning, like receptive field size, may also vary across the visual hierarchy (Vanni, Hokkanen, Werner, & Angelucci, 2020).

Electrophysiology studies provide detailed tuning curves for individual neurons and, in some cases, statistical descriptions of neural tuning within a local patch of neurons, but cannot quantify tuning across a whole map due to limited sampling. Functional MRI (fMRI) provides complementary information about spatial frequency tuning: it lacks precision at the level of individual neurons, but can systematically measure population averages across an entire map, including V1 (Broderick et al. 2022; D'Souza et al. 2016; Sasaki et al. 2001; Kay 2011) and several extrastriate maps (Aghajari, Vinke, & Ling, 2020; Farivar, Clavagnier, Hansen, Thompson, & Hess, 2017; Henriksson, Nurminen, Hyvärinen, & Vanni, 2008).

The fMRI studies above all reported that preferred spatial frequency of V1 decreases with eccentricity, but differed in their absolute estimates. Notably, two recent studies, Aghajari et al. (2020) and Broderick et al. (2022) showed good agreement in estimates of peak spatial frequency, despite employing different approaches: Aghajari et al. (2020) used a voxel-wise approach, fitting independent spatial frequency tuning curves to each voxel, whereas Broderick et al. (2022) used a map-wise approach, fitting a low-dimensional model of spatial frequency and orientation tuning across an entire visual area. This agreement suggests a potential for establishing a reliable model of spatial frequency maps in human visual cortex.

Motivated by this, we sought to replicate Broderick et al. (2022). The study used “scaled grating” stimuli, in which the local spatial frequency was inversely proportional to eccentricity. These stimuli are efficient for mapping spatial frequency preferences in parallel across the visual field, because peripheral map locations are relatively insensitive to high frequencies and foveal locations are less sensitive to low frequencies. Hence the stimuli can more efficiently sample the sensitive range of each location compared to uniform gratings. A similar set of grating stimuli was used in the ‘nsdsynthetic’ supplement to the Natural Scenes Dataset (Allen et al., 2022). We analyzed these data to ask how closely the findings of Broderick et al. (2022) replicated. In doing so, we adopted the same model parameterization used by Broderick et al., fitting the model to all the data in V1 simultaneously with 9 parameters. This parameterization imposes smooth variation in the model’s spatial frequency tuning as a function of voxel eccentricity, voxel polar angle, local stimulus orientation, and local stimulus spatial frequency. We evaluated the reliability of spatial frequency maps in V1 by comparing the parameters fit in the new dataset with the parameters fit to Broderick et al.’s data. Furthermore, we fit the same parameterized model to data from V2 and V3 in the new dataset.

By empirically assessing the reproducibility of Broderick et al.’s model in V1, and then applying the model to V2 and V3, we enhance our understanding of the organization of spatial frequency preferences within human retinotopic maps.

## 2. Materials and Methods

### 2.1. Datasets

#### 2.1.1. NSD gratings dataset

The Natural Scenes Dataset (NSD; Allen et al., 2022) is a publicly available dataset that contains extensive fMRI responses collected at ultra-high field (7T) from eight subjects. In addition to the core NSD experiment which consisted of up to 40 sessions per subject of viewing images of natural scenes, the subjects also completed one ‘NSDsynthetic’ session. This session measured brain responses to synthetic stimuli including the scaled gratings investigated in this study, as well as several other stimulus types not analyzed for this paper. In this paper, we refer to the measured responses to the scaled gratings as the ‘NSD gratings dataset’. The functional data were acquired at 7T using whole-brain gradient-echo EPI at 1.8-mm resolution. A general linear model (GLMsingle; Prince et al., 2022) was used to estimate single-trial beta weights. More details on the stimuli, data acquisition, and preprocessing can be found in the NSD paper (Allen et al., 2022) and the NSD manual (see http://naturalscenesdataset.org). The preprocessed NSD data includes GLM results in multiple formats. The GLMresults used for this study is “fithrf_GLMdenoise_RR” prepared in the "nativesurface" format. These results consist of single-trial betas (expressed in units of percent BOLD signal change) for each vertex in each subject’s native cortical surface.

##### NSD experimental design

The ‘NSDsynthetic’ session consists of 8 scans, alternating between a fixation task (detect a luminance change of the fixation dot) and a one-back task on the images (detect two consecutive trials with the same image). For the purposes of this study, we combined responses across both tasks. In the occasional cases that there were consecutive repeats of a scaled grating stimulus, the second presentation was omitted from analysis. Each stimulus was presented for 2 s, followed by 2 s of blank (uniform screen, mean luminance). Each stimulus class (see next section) was presented once in each of the 8 scans. The stimuli were displayed within an 8.4° x 8.4° square centered on the screen against a gray background. Throughout the experiment, a circular anti-aliasing mask with a small semi-transparent fixation dot (0.1° radius) was present in the center of the screen. The anti-aliasing mask crops out the central region in which the spatial frequency is too high to render without aliasing. The size of the anti-aliasing mask is spatial frequency dependent, ranging from 0.02° radius for the scaled grating with the lowest frequency to 0.5° for the scaled grating with the highest frequency. Our analyses excluded vertices if the pRF center was within the largest antialiasing mask for any stimulus.

##### NSD spatial frequency stimuli

The NSD synthetic experiment contained a total of 284 images including the 112 scaled grating stimuli used in this study. These stimuli were designed to take advantage of the known inverse relationship between preferred spatial frequency and eccentricity. Scaled gratings in NSD were constructed in the same manner as the Broderick et al. (2022), according to the equation:

(1) $$I(r,\theta )\;=\;cos(\omega_{r}ln(r)+\omega_{a}\theta +\phi )$$

The pixel intensities in the image, I, at polar coordinates (r,θ) are specified in terms of two frequency parameters, $\omega_{a}$ (angular frequency in cycles per revolution around the image) and $\omega_{r}$ (radial frequency specifying radians per unit increase in ln(r)), and one phase parameter ϕ (in radians). The local spatial frequency $\omega_{l}$ depends only on the pixel eccentricity, r, and the frequency vector $\left(\omega_{r},\omega_{a}\right)$:

(2) $$\omega_{l}(r)=\frac{\sqrt{\omega_{r}^{2}+\omega_{a}^{2}}}{r}$$

(See also Supplement section 1.1 in Broderick et al.(2022))

Likewise, the local orientation $\theta_{l}$ depends only on the pixel polar angle, θ, and the frequency vector $\left(\omega_{r},\omega_{a}\right)$:

(3) $$\theta_{l}(\theta )\;=\;\theta +tan^{-1}\left(\frac{\omega_{a}}{\omega_{r}}\right)$$

**Figure 1.**
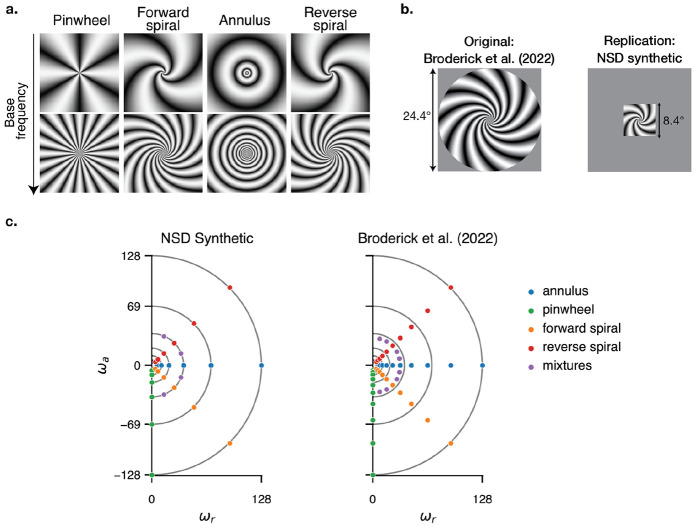
Stimuli for the two studies. a) Example stimuli with two different base frequencies (6 and 20) and four orientations. b) Stimulus size and aperture (circular vs square) difference between the two studies. Other differences between the studies are listed in Table 1. c) Frequency vectors $\left(\omega_{r},\omega_{a}\right)$ of the stimulus classes in the two studies. The gridlines are the same in two plots to facilitate comparison of base frequencies.

Each stimulus class was defined by its frequency vector $\left(\omega_{r},\omega_{a}\right)$. There were 28 stimulus classes in the experiments, with 4 exemplars per class defined by their phase (0, 1.57, 3.14, 4.71 radians). The 28 stimulus classes are organized into four shapes and “mixtures”, which are intermediate between the other four shapes (Figure 1a):

1. Pinwheels:
$$\omega_{r}=0,\;\omega_{a}\in \{\text{-}6,\text{-}11,\text{-}20,\text{-}37,\text{-}69,\text{-}128\}$$
2. Annuli:
$$\omega_{a}=0,\;\omega_{r}\in \{6,11,20,37,69,128\}$$
3. Forward spirals:
$$\omega_{r}=-\omega_{a}\in \{4,7,14,26,49,91\}$$
4. Reverse spirals:
$$\omega_{r}=\omega_{a}\in \{4,7,14,26,49,91\}$$
5. Mixtures:
$$\left(\omega_{r},\;\omega_{a}\right)\in \{(14,\text{-}34),(34,\text{-}14),(34,14),(14,34)\}$$

The frequency vectors were selected so that the base frequencies (vector length of $\left(\omega_{r},\omega_{a}\right)$) matched across shapes (i.e., the stimulus coordinates lie on circles centred at the origin in Figure 1c). A constraint on the parameters is that $\omega_{a}$ are integers, as non-integer circular frequencies result in edge artifacts. Because of this constraint, there are slight discrepancies in base frequencies between the pinwheel/annulus stimuli and the forward/reverse spiral stimuli, as in Broderick et al. (2022).

##### NSD retinotopy data and visual areas

Retinotopy data were acquired from NSD subjects in a separate session. Full details are available in the original paper (Allen et al., 2022). The mapping stimuli were slowly moving apertures filled with dynamic colorful textures, extending up to 4.2 deg eccentricity. A small fixation dot (0.1 deg in radius) was superimposed on the stimuli at the screen center. A total of six scans (three with bar apertures, three with wedge/ring apertures) were conducted. Population receptive field (pRF) models were fit to the time series of each voxel from each subject. Retinotopic ROIs were hand-drawn by the NSD authors based on the analyzed pRF data. Here, we used the pRF solutions and ROIs on the individual subject native surface, as made available by the NSD.

#### 2.1.2. Broderick et al. dataset

We compared the results from NSD gratings dataset to those from Broderick et al. (2022), which measured BOLD responses to the scaled gratings from 12 subjects. Briefly, functional data were acquired at a 3T scanner with a spatial resolution of 2 mm. The response amplitudes to the stimuli were estimated for each surface vertex for each stimulus class, with 100 bootstraps across runs using GLMdenoise (Kay, Rokem, Winawer, Dougherty, & Wandell, 2013). The GLMdenoise algorithm differs slightly from the GLMsingle tool used for the NSD in that it estimated beta weights per stimulus type, not per trial, and in several other minor ways. We re-analyzed the data from Broderick et al to ensure that we used the same computational methods for the two datasets. The re-analysis used the median beta weight per vertex per condition (median values across bootstrapped scans). These were made publicly available by the authors (https://archive.nyu.edu/handle/2451/63344). More detailed description of the Broderick dataset is provided in the original study.

##### Broderick et al. experimental design

Subjects participated in 12 scans, performing a one-back task on an alternating stream of black and white digits at fixation, while viewing the scaled gratings. Stimuli were constrained to a circular aperture up to 12° radius, with an antialiasing mask at the fixation center (0.96° radius). Each trial consisted of eight images with different phases from the same stimulus class, 300 ms per image with 200 ms ISI, for a total of 4 s per trial. The stimulus classes were presented in pseudo-randomized order.

#### 2.1.3. Comparison of experimental design

The scaled grating stimuli used in both NSD gratings and Broderick et al. datasets were generated in the same manner. The stimulus sets were constructed such that the maximum and minimum base frequencies were the same (Figure 1c). However, there were several design differences between the two experiments (Table 1) in addition to the differences in MR acquisition (e.g., 7T vs 3T) and analysis pipeline already noted (e.g. preprocessing methods, and GLMDenoise vs GLMSingle).

### 2.2. One-dimensional spatial frequency model

We fit a log-normal tuning function to the mean response amplitudes of data binned in 0.5° steps from 0.5° to 4°. We excluded vertices whose mean response amplitude across all stimuli was negative. We express spatial frequency tuning in terms of spatial period (deg per cycle, the reciprocal of spatial frequency) rather than frequency, because the period is expected to grow linearly with eccentricity, and it is easier to assess by visual inspection how well data conform to a line than to how well data conform to an inverse linear function. The log Gaussian tuning function for eccentricity bin b is expressed as:

(3) $${\hat{\beta }}_{b}\left(p_{l}\right)=A_{b}\cdot exp\left(\frac{{\left(log_{2}(p_{l})-log_{2}(p_{b})\right)}^{2}}{2\sigma_{b}^{2}}\right)$$

where $A_{b}$ is the response gain, $p_{b}$ is the preferred spatial period, $p_{l}$ is the local spatial period (at eccentricity b) and $\sigma_{b}$ is the bandwidth in octaves. The tuning curve was fit separately for each subject to data averaged across vertices and across the four primary stimulus shapes (annuli, pinwheels, spirals) for each base frequency. Hence for this model, the response of a vertex depends only on the local spatial frequency, not orientation.

#### 2.2.1. Precision weighted average

We summarized the 1D model results across subjects using a precision-weighted average. Each subject’s contribution to the average was weighted by the reciprocal of the variance,

(4) $$\bar{p_{b}}=\frac{\sum_{s=1}^{8}\frac{p_{\left(b,s\right)}}{\sigma_{s}^{2}}}{\sum_{s=1}^{8}\frac{1}{\sigma_{s}^{2}}}$$

where $p_{\left(b,s\right)}$ is the peak spatial period for eccentricity bin b and subject s, and $\sigma_{s}^{2}$ is the variance for subject s, defined as follows:

(5) $$\sigma_{s}^{2}=\frac{1}{m}\sum_{v=1}^{m}\sigma_{v}^{2},\;\sigma_{v}^{2}=\frac{1}{n}\sum_{i=1}^{n}\sigma_{vi}^{2}$$

The subject variance, $\sigma_{s}^{2}$ is the variance averaged across the m vertices in a map. The variance of each vertex, $\sigma_{v}^{2}$, is the variance averaged across 24 stimulus classes, with the variance for the $i^{\text{th}}$ stimulus class, $\sigma_{vi}^{2}$, computed across beta weights for the 8 repeated images within each stimulus class i. The precision-weighted average assigns greater weight to data with high signal-to-noise ratio, while still incorporating all of the data. Because each subject is assigned a single precision value, the weight of each subject is the same for every eccentricity bin.

### 2.3. Two-dimensional spatial frequency model

**Figure 2.**
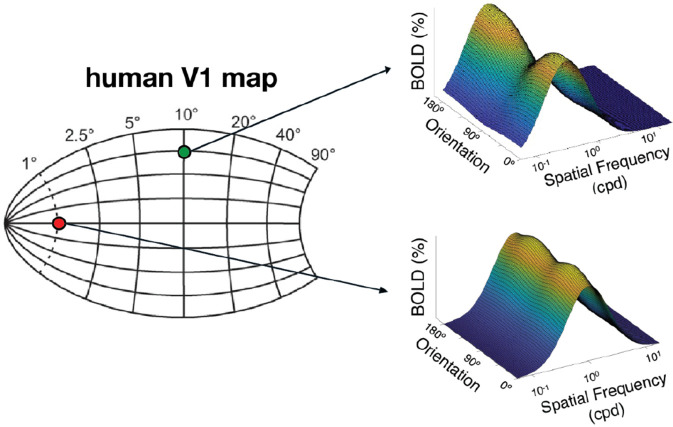
2-D model schematic. Each location in V1 (left) corresponds to a visual field position, specified by eccentricity and polar angle. At each location, the model (right) has a 2D tuning curve, predicting the BOLD amplitude as a function of local spatial frequency and orientation. The schematic shows example tuning curves for two locations in the visual field (red dot: 1° eccentricity on the horizontal meridian; green dot: 10° eccentricity near vertical meridian). The peak spatial frequency is higher for the more foveal location and the orientation modulation is larger for the other location.

Since the one-dimensional tuning curve model cannot measure the effect of stimulus orientation and retinotopic angle, Broderick et al. (2022) further developed a two-dimensional model for individual vertex responses. The model takes inputs as the vertex eccentricity and polar angle, and the stimulus spatial frequency and orientation at that location, and predicts the BOLD response as output (Figure 2). We can thus express the model response to a given stimulus for vertex v as follows:

(6) $${\hat{\beta }}_{v}\left(p_{l},\theta_{l}\right)=A_{v}\cdot exp\left(\frac{{\left(log_{2}(p_{l})-log_{2}(p_{v})\right)}^{2}}{2\sigma^{2}}\right)$$

Here $p_{v}$ is preferred period of the vertex and σ is the bandwidth, assumed to be equal across the map. $p_{l}$ is the local spatial period (degree/cycle) and $\theta_{l}$ is the local orientation (radians). To understand how spatial frequency preferences vary across vertices within a visual area, $p_{v}$ is forced to linearly vary as a function of vertex eccentricity $r_{v}:p_{v}=a\cdot p_{v}+b$. Furthermore, $p_{v}$ is modulated by orientation. Specifically, $p_{v}$ is expressed as:

(7) $$p_{v}=\left[ar_{v}+b\right]\left[1+p_{1}cos\left(2\theta_{l}\right)+p_{2}cos\left(4\theta_{l}\right)+p_{3}cos\left(2\left(\theta_{l}-\theta_{v}\right)\right)+p_{4}cos\left(4\left(\theta_{l}-\theta_{v}\right)\right)\right]$$

Each of the parameters $p_{i}$ controls how $p_{v}$ depends on a particular aspect of orientation. Specifically, for positive values of $p_{i}$, preferred period is larger for some orientations than others

1. $p_{1}$: Vertical > horizontal (“horizontal effect”)
2. $p_{2}$: Cardinal > oblique (“oblique effect”)
3. $p_{3}$: Annulus > pinwheel (“radial effect”).
4. $p_{4}$: Non-spirals (annuli/pinwheels) > spirals (“spiral effect”).

Those four $p_{i}$ can be grouped into absolute orientation effects ($p_{1}$ and $p_{2}$) and relative orientation effects ($p_{3}$ and $p_{4}$). Absolute orientation effects depend on the local orientation $\theta_{l}$ but not on the vertex’s retinotopic angle $\theta_{v}$. Relative orientation effects depend on the difference between the local orientation $\theta_{l}$ and a vertex’s retinotopic angle $\theta_{v}$.

Similarly, we allow the amplitude of the BOLD response to vary with stimulus orientation:

(8) $$A_{v}\;=\;1+A_{1}cos\left(2\theta_{l}\right)+A_{2}cos\left(4\theta_{l}\right)$$

**Figure 3.**
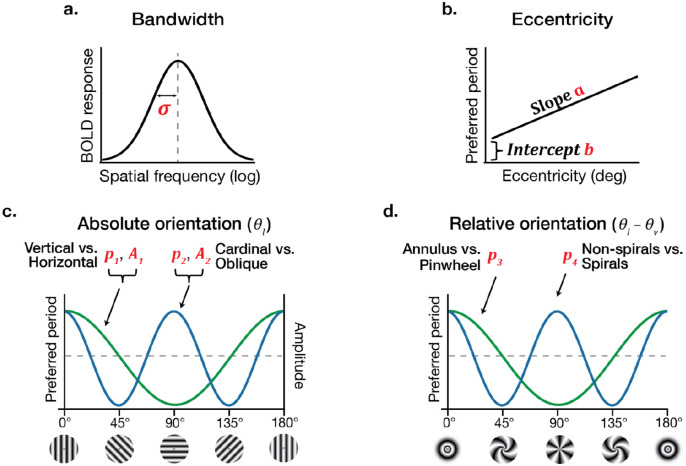
Schematic representation of the 9 parameters in the two-dimensional model. a) Bandwidth σ. In this study, σ is assumed to be consistent across eccentricity, retinotopic location, and local stimulus properties. b) Eccentricity effect (Slope a and intercept b). Preferred period is assumed to be an affine function of eccentricity. c) Four parameters related to absolute orientation. $p_{1}$ and $p_{2}$ represent the orientation effect on preferred period, whereas $A_{1}$ and $A_{2}$ represent the effect on the gain of the BOLD responses. $p_{1}$ and $A_{1}$ are positive when the presented orientation is vertical vs. horizontal. $p_{2}$ and $A_{2}$ are positive when the orientation is cardinal vs. oblique. d) Two parameters on the stimulus orientation relative to retinotopic angle. $p_{3}$ is positive when the relative orientation is annulus (radial) vs. pinwheel (tangential) and $p_{2}$ is positive when the orientation is spirals vs. non-spirals.

Following Broderick et al. (2022), we do not allow amplitude to vary with relative orientation, as inclusion of these parameters did not increase cross-validated model accuracy. We summarize the 9 model parameters in Figure 3.

We fit the two-dimensional model to all data within an entire map at once, excluding vertices whose pRF centers were more than one pRF size outside a 4.2° radius, and vertices whose averaged responses across the 28 stimulus classes were negative.

Following Broderick et al., the loss function was weighted by vertex precision, giving less weight to noisier vertices. The loss for vertex v is expressed as follows:

(9) $$L_{v}\left(\beta_{v},{\hat{\beta }}_{v}\right)=\frac{1}{\sigma_{v}^{2}}\sum_{i=1}^{n}\frac{1}{n}{\left(\frac{\beta_{iv}}{{\left\|\beta_{v}\right\|}_{2}}-\frac{{\hat{\beta }}_{iv}}{{\left\|{\hat{\beta }}_{v}\right\|}_{2}}\right)}^{2}$$

Here the loss, $L_{v}$, is the weighted, L2-normalized mean-squared error between beta weights from the GLM and predicted responses from the model. The 28 stimulus classes are indexed by i. $\beta_{iv}$ is the beta estimate of voxel v to stimulus class i from the GLM. ${\hat{\beta }}_{iv}$ is the model prediction for stimulus class i and vertex v. ${\left\|\beta_{v}\right\|}_{2}$ is the L2-norm of $\beta_{v}$ across the 28 stimulus classes, and $\sigma_{v}^{2}$ is the vertex variance, computed by taking the mean of the variance computed separately for each of the 28 stimulus classes (8 trials per class).

All model fitting procedures, including the one-dimensional tuning curves and the 2D model, were written in PyTorch (Paszke, Gross, Massa, & Lerer, 2019) using the Adam optimization algorithm (Kingma & Ba, 2014).

## 3. Results

The ‘nsdsynthetic’ supplement to the Natural Scenes Dataset (Allen et al., 2022) included scaled grating stimuli, similar to those used by Broderick et al. (2022), but with several differences in the stimulus and experiment details (Table 1). Notably, due to constraints in the display setup, NSD stimulated less of the visual cortex than Broderick et al (3x lower eccentricity extent) with fewer trials (224 vs 733). On the other hand, the NSD used smaller voxels, higher field strength, and smaller image pixels (enabling measurements closer to the fovea). Before fitting the full 2D model, we did a coarse analysis on data binned across vertices (within an eccentricity band) and binned across stimulus orientation. This analysis fits 1D spatial frequency tuning curves to data averaged within eccentricity bins.

To preview the results, we first show with the 1D model fits that NSD has good quality spatial frequency data, both in V1 and in extrastriate maps (V2 and V3). The V1 data are in reasonably good agreement with Broderick et al. We then examine the V1 data in more detail, comparing 2D model fits between NSD and Broderick et al. Most but not all parameters are in good agreement between the two datasets. Finally, we report the 2D model fits in NSD V2 and V3. The biggest difference between tuning in these areas in V1 is larger bandwidth in the extrastriate maps.

### 3.1. 1D model: NSD-synthetic has good responses to scaled grating stimuli

**Figure 4.**
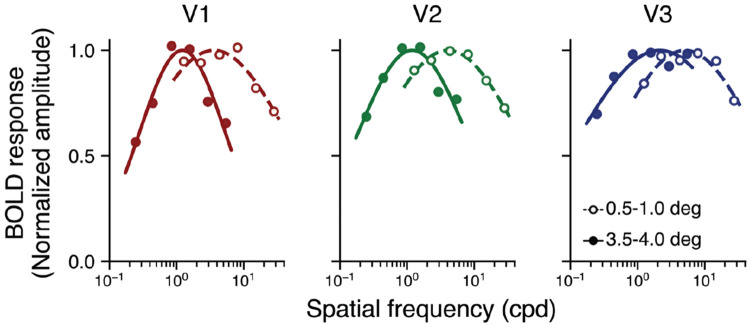
Example spatial frequency tuning curves for NSD. Each panel shows data points binned by eccentricity fit by log Gaussian tuning curves in an example NSD subject (subj08). Data are plotted for two eccentricity bins. Data and tuning curves are scaled so that the maximum value of each peak of the tuning curves is 1.

The 1D tuning curve analysis shows high quality data in NSD. We first consider an example dataset from one subject (Figure 4). For this subject, the BOLD responses in V1, V2, and V3 were all well fit by log-Gaussian tuning curves, similar to Broderick et al. As expected, the preferred spatial frequency declined with eccentricity: in all three visual maps, the tuning curves peaked at about 2 to 3 cpd in the foveal bins (0.5 to 1.0 deg), and about 1 cpd in the parafoveal bins (3.5 to 4 deg). The bandwidth was also narrower in V1 than in V2 and V3.

Next we quantified these observations across all subjects and eccentricity bins, and found that the data show two expected systematic patterns, in general agreement with Broderick et al.

**Figure 5.**
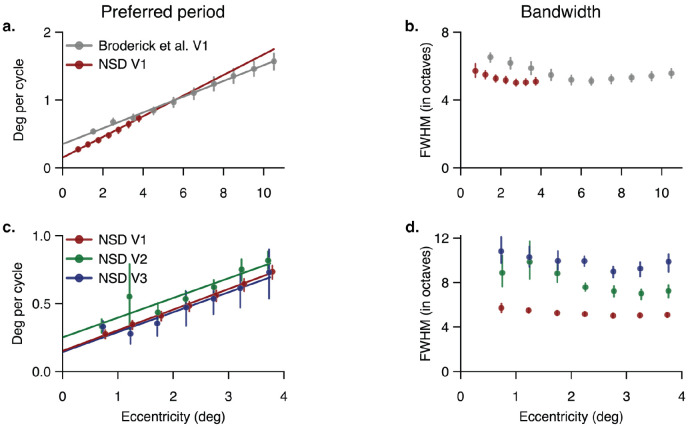
Preferred period (left column) and bandwidth (right column) for Broderick et al. and NSD, estimated by 1D model. The top row (a,b) shows V1 properties in Broderick et al and NSD (“Replication”) and the bottom row (c,d) shows results from V1, V2 and V3 in NSD, with NSD V1 data replotted (“Extension”). a,c) Preferred period of tuning curves as a function of eccentricity. b,d) Full-width at half max (in octaves) of tuning curves as a function of eccentricity. Circles indicate precision-weighted means at each eccentricity bin and lines are the best linear fits to the data points. The error bars represent 68% bootstrapped confidence intervals across subjects.

First, in V1, the preferred period (reciprocal of preferred spatial frequency), increases with eccentricity, and is well described by a straight line with a positive intercept (Figure 5a). The preferred period in NSD is close to, but slightly lower than, that in Broderick et al., an observation we return to in the more detailed 2D models in section 3.2. There is good agreement in preferred period between NSD observers, as evident by the small confidence intervals in Figure 5a.

Second, the bandwidth of the tuning curves in NSD V1 is large, about 5 octaves (full width at half max) for eccentricities over 2 deg, and a little higher near the fovea. This is similar to the pattern in Broderick et al’s dataset (Figure 5b), although the bandwidth in NSD is slightly lower.

We also examined spatial frequency tuning in V2 and V3 in NSD, which was not reported by Broderick et al. We find that V2 and V3 show reliable increases in preferred period with eccentricity (Figure 5c). As in V1, these data are well fit by lines with positive intercepts. The agreement across NSD subjects in V2 and V3 is not quite as high as in V1, as indicated by the larger error bars. The tuning width of NSD substantially increases from V1 to V2, and slightly more from V2 to V3 (Figure 5d), paralleling the tendency for receptive field size to increase along the cortical hierarchy (Dumoulin & Wandell, 2008). Averaged across eccentricity bins, the tuning width (full-width at half max in octaves) in NSD increases from about 5 in V1 to 8 in V2 to 10 in V3 (More precise estimates are reported for the 2D model in subsequent sections).

### 3.2. A comparison of 2D model fits for NSD V1 vs Broderick et al. V1

To compare the NSD and Broderick et al. results more quantitatively, we used the same two-dimensional model from Broderick et al. and fit it to each NSD subject’s data. The model predicts the BOLD response amplitude at each location in the V1 map as a function of the local spatial frequency and orientation in the stimulus, where “local” means the value at the pRF center. The model predictions differ across locations (i.e., vertices) but in highly constrained ways, enabling us to make reasonably good predictions for all stimulus orientations, spatial frequencies, and map locations with only 9 free parameters. Thus, the model provides a concise description of spatial frequency preferences in an entire visual field map. To ensure that any differences in parameter estimates are due to differences in the datasets rather than the analysis pipeline, we re-analyzed the Broderick et al. dataset using the same code applied to the NSD.

Below, we compare the 9 parameter estimates between NSD and Broderick et al. As in Broderick et al., we combined model parameters across subjects using a precision-weighted average (see Methods). To preview the results, we find that parameters which pertain to eccentricity and to absolute orientation (stimulus orientation irrespective of pRF location) generally replicate well, whereas the two parameters that pertain to stimulus orientation relative to the pRF polar angle do not.

#### 3.2.1. V1 spatial frequency bandwidth is similar between NSD and Broderick et al.

**Figure 6.**
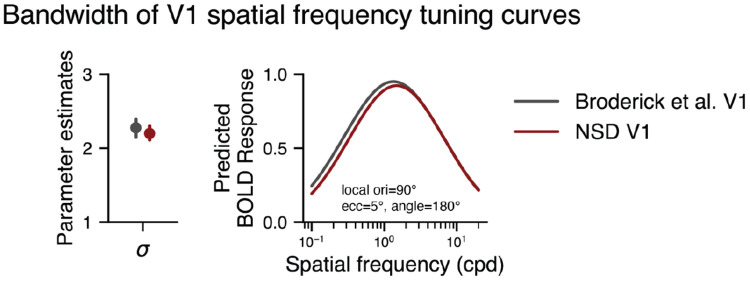
Bandwidth of V1 spatial frequency tuning curves (“Replication”). The left plot shows the estimated sigma parameter for the two datasets, Broderick et al. and NSD. The circles represent precision-weighted averages of bandwidth, where bandwidth is one standard deviation of the log Gaussian tuning curve, in octaves. The error bars indicate 68% bootstrapped confidence intervals across subjects. The right panel shows example model responses, averaged across subjects, assuming local orientation at 90° and a voxel’s pRF eccentricity and angle at 5° and 180°, respectively.

Because the 1D model showed that the bandwidth was relatively stable across eccentricity bins, the 2D model assumed a single bandwidth (in octaves, not in degrees). The bandwidth parameter σ was the same for NSD and Broderick et al., 2.3±0.1 and 2.2±0.1 octaves, respectively (Figure 6 left). This value is the standard deviation of the Gaussian tuning curve fit to log spatial frequency data. A standard deviation of 2.2 corresponds to a full-width-at-half-max of 5.2, nearly identical to the value of 5.3 estimated from the 1D model for V1 (Figure 5b). To visualize model tuning curves with the fit widths, we plot example model tuning curves (Figure 6 right).

#### 3.2.2. V1 spatial frequency tuning as a function of eccentricity is similar for NSD and Broderick et al.

**Figure 7.**
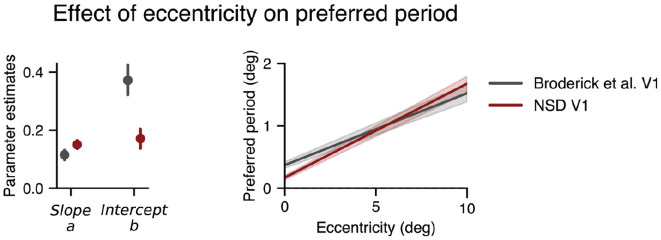
Eccentricity effect of V1 in Broderick et al and NSD (“Replication”). The left panel shows the estimated slope and intercept from the two-dimensional model. The circles represent precision-weighted averages. The error bars indicate 68% bootstrapped confidence intervals across subjects. The right panel shows the predicted preferred period as a function of eccentricity based on the values shown left, with local orientation at 90° and a voxel’s retinotopic angle at 180°.

Next we compared the effect of eccentricity on preferred period between the two datasets in V1. The slope parameter a showed good agreement: Broderick et al. had an average slope of 0.12±0.02, while NSD V1 had a slope of 0.14±0.01 (Figure 7 left). However, there was a noticeable difference in the intercepts b, with NSD intercept half of Broderick et al.’s. The lower intercept for NSD may be due to the differences in experimental details, such as the voxel size or stimulus size. Nonetheless, both intercept values were reliably above zero, indicating that spatial period is not proportional to eccentricity. Moreover, across eccentricities, the lower intercept but slightly higher slope in NSD compared to Broderick et al. combine to produce similar tuning across the central 10 deg of eccentricity (Figure 7 right).

#### 3.2.3. Both V1 datasets show higher preferred period for vertical than horizontal and for oblique than cardinal

**Figure 8.**
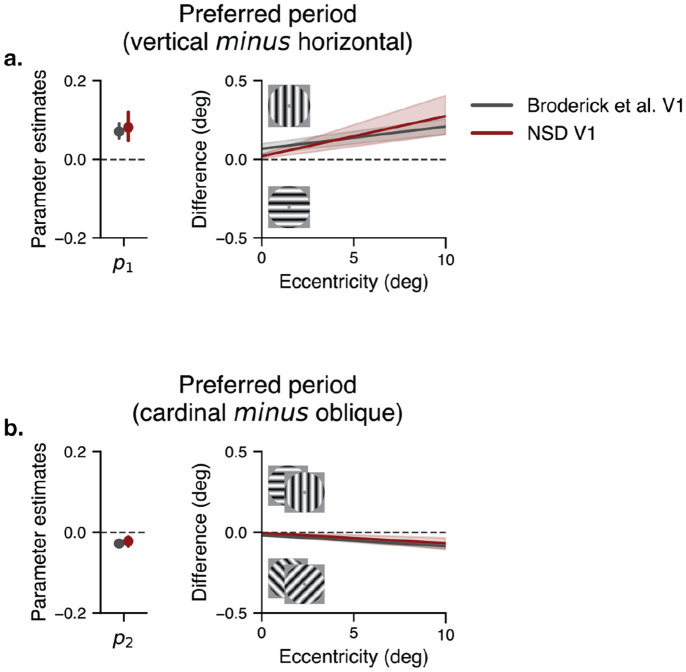
Absolute orientation effect on preferred period of V1 in Broderick et al. and NSD (“Replication”). For all the panels a and b, the left column shows the estimated parameter values. The circles and the error bars represent precision-weighted averages and 68% bootstrapped confidence intervals across subjects. The right column shows the difference in preferred period at local orientation 90° and retinotopic angle 180°. The difference was calculated between the two orientations on which each p parameter depends. a) shows $p_{1}$ value in the left panel, and the difference in preferred period between vertical and horizontal orientations in the right panel. b) shows $p_{2}$ in the left panel, and the difference in preferred period between cardinal and oblique orientations in the right panel.

Both datasets show that stimulus orientation modulates spatial frequency tuning. This is shown by the estimates of parameters $p_{1}$ and $p_{2}$. The estimates are positive for $p_{1}$, indicating a larger preferred period for vertical than horizontal orientations (Figure 8a): 0.07±0.02 and 0.08±0.04 for Broderick et al. and NSD, respectively. The $p_{2}$ parameters are negative, indicating a larger preferred period for oblique than cardinal orientations (Figure 8b): −0.03±0.006 and −0.02±0.01 for Broderick et al. and NSD, respectively. The negative value of $p_{2}$ aligns with the well-documented “oblique effect” in visual perception: poorer discrimination and detection for oblique than cardinal orientations (Appelle, 1972; Mach, 1861). It is not clear whether positive value of $p_{1}$ has a psychophysical correlate, as performance differences between horizontal and vertical stimuli are inconsistent across studies (for example, Berkley, Kitterle, & Watkins, 1975; Bulens, Meerwaldt, & Van der Wildt, 1988; Campbell, Kulikowski, & Levinst 1966; Long & Tuck, 1991). Both of these orientation effects are modest in size, indicating variation in preferred period as a function of orientation of no more than about 12%.

#### 3.2.4. Both datasets show higher BOLD amplitude for vertical than horizontal in V1

**Figure 9.**
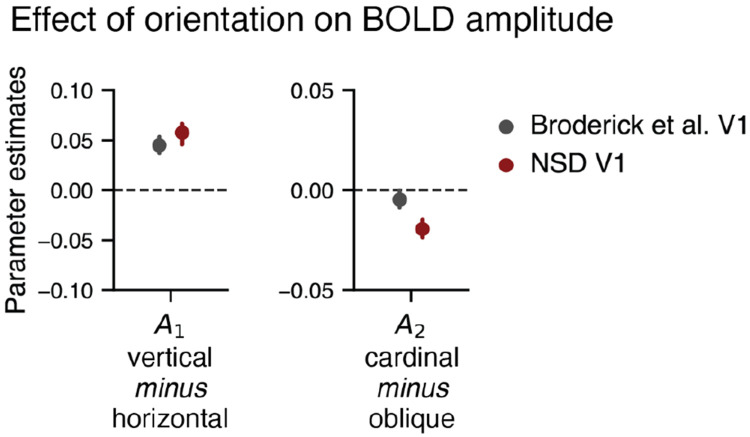
Absolute orientation effect of V1 on BOLD amplitude in Broderick et al and NSD (“Replication”). The panel shows two estimated parameters ($A_{1}$, $A_{2}$). The circles and the error bars represent precision-weighted averages and 68% bootstrapped confidence intervals across subjects.

The parameters discussed so far pertained to spatial frequency tuning in V1, independent of amplitude. Here we consider the effect of stimulus orientation on amplitude, independent of period. A small but consistent orientation effect on the BOLD responses is observed in both of the datasets. Specifically, in both Broderick et al. and NSD, the $A_{1}$ parameter is positive, indicating higher BOLD amplitude for vertical than horizontal orientations (Figure 9 left). The two parameter values are about the same, Broderick at 0.04 (0.036, 0.053 68% CI) and NSD at 0.06 (0.046, 0.066 68% CI), meaning that the stimulus orientation modulates the preferred period up or down by 5 or 6%.

The two datasets diverge in their estimates for the parameter $A_{2}$ (Figure 9 right). NSD has a negative $A_{2}$, meaning higher amplitude for oblique than cardinal orientations whereas the estimate from Broderick et al is slightly negative but close to 0 .

#### 3.2.5. Inconsistent effects of relative orientation on preferred period

**Figure 10.**
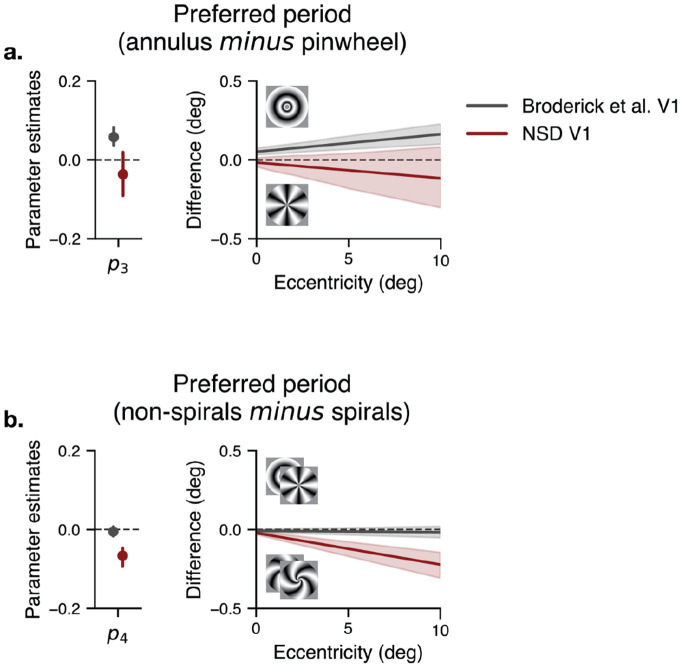
Relative orientation effect on preferred period of V1 in Broderick et al. and NSD (“Replication”). For all the panels a and b, the left column shows the estimated parameter values, whereas the right column shows the difference in preferred period at local orientation at 90° and retinotopic angle at 180°. The difference was calculated between the two orientations on which each p parameter depends. In the left column panels, the parameters are labeled after the orientation with a greater preferred period when the values are negative. a) The results of $p_{1}$ which depends on circular (“annulus”) versus radial (“pinwheel”) orientation. b) The results of $p_{2}$ that depends on non-spiral (“annulus” and “pinwheel”) versus spiral (“forward spiral” and “reverse spiral”) orientation. The dots and the error bars represent precision-weighted means and 68% bootstrapped confidence intervals across subjects.

The last two model parameters are $p_{3}$ and $p_{4}$, corresponding to orientation relative to retinotopic angle. There were no consistent effects for these parameters. Broderick et al. estimated a positive $p_{3}$ value, meaning preferred period was higher for annuli than pinwheels, but NSD did not (Figure 10a). The difference might be explained by an interaction with eccentricity. The NSD coverage was more foveal (0.5 to 4.2 deg) than Broderick et al. (1 to 12 deg) and in another study (Himmelberg, VSS 2024), the difference in spatial frequency tuning for pinwheels vs annuli reversed with eccentricity: Near the fovea, the peak period was higher for radial patterns, but at 3 deg and beyond, the peak period was higher for annuli.

The parameter $p_{4}$ was negative in NSD but 0 in Broderick et al. (Figure 10b). A negative $p_{4}$ indicates a higher preferred period for spirals than non-spirals (annuli or pinwheels).

#### 3.2.6. Replication summary

**Figure 11.**
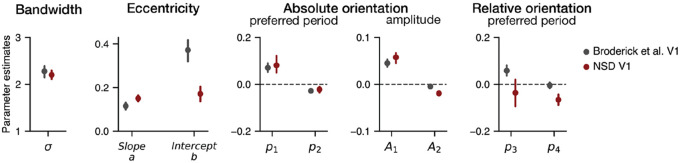
2D parameter estimates for NSD and Broderick et al. for V1.

In sum, the 2D model of V1 spatial frequency tuning in the NSD synthetic dataset reproduced several, though not all, of the effects reported previously by Broderick et al. A summary of all 9 parameters from the two datasets is plotted in Figure 11. The bandwidth, effects of eccentricity (especially the combined effect of slope and intercept), and effects of absolute orientation were similar in the two datasets (with the exception of $A_{2}$, amplitude of cardinal vs oblique).

### 3.3. The extension of 2D model analysis to V2 and V3

We now extend our analysis by applying the two-dimensional model to extrastriate areas V2 and V3 from NSD. By comparing the 9 parameter estimates across the visual areas, our goal is to identify systematic changes in spatial frequency preferences along the visual hierarchy. To preview the results, we find that the bandwidth of spatial frequency tuning increases from V1 to extrastriate areas, whereas the preferred period does not differ much. Moreover, the oblique effects observed in V1 persist in V2 and V3. Specifically, both the preferred period and the amplitude are higher for oblique orientations than for cardinal orientations in V2 and V3.

#### 3.3.1. Increasing bandwidth from V1 to extrastriate areas

**Figure 12.**
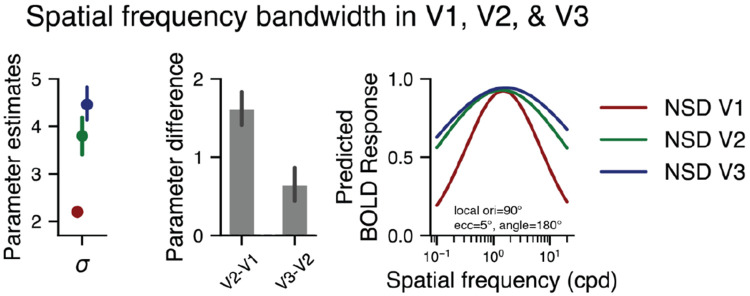
Estimated bandwidth values (left) and spatial frequency tuning curves (right) of V1, V2 and V3 in NSD (“Extension”). NSD V1 data from figure 6 is replotted as a reference. The circles represent precision-weighted averages of bandwidth, and the error bars indicate 68% bootstrapped confidence intervals across subjects. The middle panel shows within-subject differences of the parameter along visual hierarchy. The right panel shows the example model responses, averaged across subjects, assuming local orientation at 90° and a voxel’s pRF eccentricity and angle at 5° and 180°, respectively.

There was a large increase in bandwidth from V1 to V2 and an additional but smaller increase from V2 to V3. The bandwidth parameter σ was 2.2±0.1 octaves for V1, 3.8±0.4 octaves for V2, and 4.5±0.3 octaves for V3 (Figure 12 left). This corresponds to a full-width-at-half-max of 5.2, 9.0, and 10.6 (V1, V2, and V3). This indicates a 70% increase in bandwidth from V1 to V2, and 100% increase from V1 to V3. To show that the increases in bandwidth from V1 to V2 and from V2 to V3 are reliable within subjects, we plot the parameter differences in the middle panel of Figure 12. These differences are large relative to the confidence interval of the differences. Model tuning curves are visualized in the right panel of Figure 12 across the three areas.

#### 3.3.2. Preferred period does not vary systematically across the V1 to V3 maps

**Figure 13.**
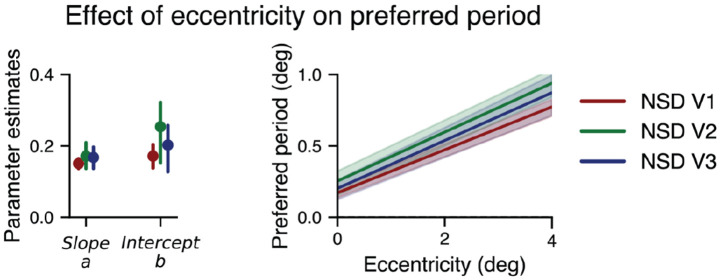
Eccentricity effect on preferred period of V1, V2, and V3 in NSD gratings dataset, with V1 data from Figure 7 replotted as a reference. The left panel shows the estimated slope and intercept values from the two-dimensional model. The circles represent precision-weighted averages. The error bars in both left and right panels indicate 68% bootstrapped confidence intervals across subjects. The right panel shows the predicted preferred period as a function of eccentricity based on the values shown left, with local orientation at 90° and a voxel’s retinotopic angle at 180°.

In contrast to the effect of bandwidth, spatial frequency tuning as a function of eccentricity is similar across the V1 to V3 maps. Within each map, the line fit to preferred period vs eccentricity has a positive y-intercept (0.17 to 0.25 deg per cycle at the fovea), and a positive slope (0.15 to 0.17) (Figure 13, left). The lines do not show a systematic increase in period with visual areas, with V3 numerically between V1 and V3, though the differences between areas are not large or robust.

#### 3.3.3. All three maps have higher preferred period for vertical than horizontal and for oblique than cardinal orientations

**Figure 14.**
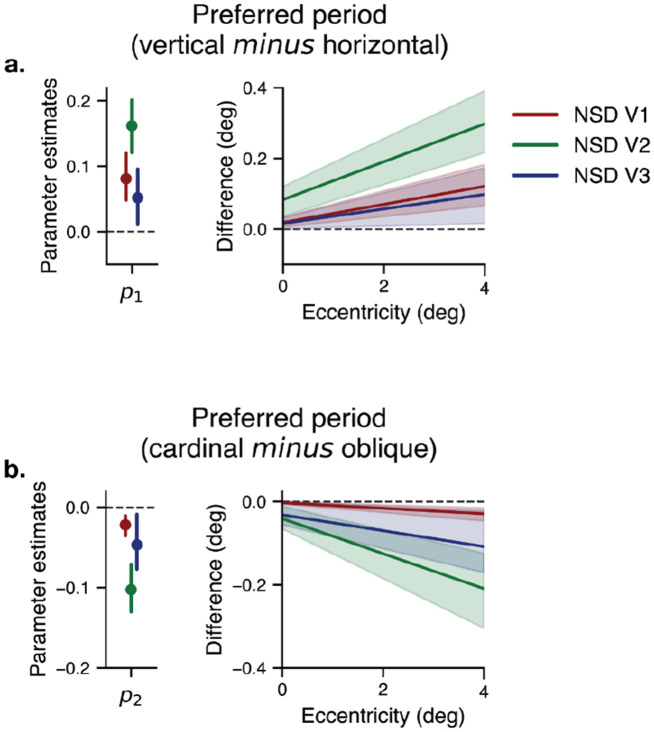
Absolute orientation effect on preferred period of V1, V2 and V3 in NSD (“Extension”), with NSD V1 data from Figure 8 replotted. For all the panels a and b, the left column shows the estimated parameter values. The circles and the error bars represent precision-weighted averages and 68% bootstrapped confidence intervals across subjects. The right column shows the difference in preferred period at local orientation 90° and retinotopic angle 180°. The difference was calculated between the two orientations on which each p parameter depends. a) shows the difference in preferred period between vertical and horizontal orientations. b) shows the difference in preferred period between cardinal and oblique orientations.

In V1, preferred period was slightly but reliably higher for vertical than horizontal orientations. The same pattern was observed in V2 and V3, although the effect is weak in V3. These patterns are summarized by the $p_{1}$ parameter estimates, which was 0.08±0.03 for V1, 0.16±0.04 for V2, and 0.05±0.04 for V3 (Figure 14a). Additionally, all the early visual areas showed negative values for parameter $p_{2}$, indicating that preferred period is higher for oblique than cardinal orientations (Figure 14b). Both of these two orientation effects are largest in V2.

#### 3.3.4. Effects of absolute orientation on amplitude are similar across V1, V2, and V3

**Figure 15.**
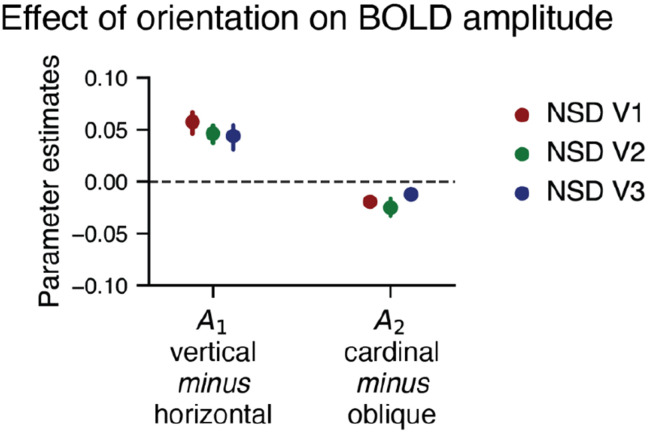
Absolute orientation effect on BOLD amplitude in NSD V1, V2, and V3 (“extension”). The panel shows two estimated parameters ($A_{1}$, $A_{2}$) for each visual area. The circles and the error bars represent precision-weighted averages and 68% bootstrapped confidence intervals across subjects.

Next, we examined the effect of stimulus orientation of extrastriate areas on BOLD amplitude. The parameter $A_{1}$, which contrasts amplitude for vertical vs horizontal, was positive for V1, V2, and V3 (Figure 15). The parameter $A_{2}$, which measures amplitude for oblique compared to cardinal, was negative for all three maps, indicating higher amplitude for oblique than cardinal orientations. Thus, in all three maps in NSD, for oblique stimuli, the preferred period is higher ($p_{2}$) and the amplitude is higher ($A_{2}$).

Note that all of the amplitude effects, even when statistically reliable, are small: for example, the $A_{2}$ parameter for V2 is about −0.02±0.01, meaning that the predicted BOLD responses to oblique gratings and cardinal gratings differ by about 4%. More concretely, if the mean BOLD response in a voxel were, say, 1% signal change, then the model would predict 1.02% for oblique and 0.98% for cardinal.

#### 3.3.5. No systematic effects of relative orientation on preferred period across V1, V2 and V3

**Figure 16.**
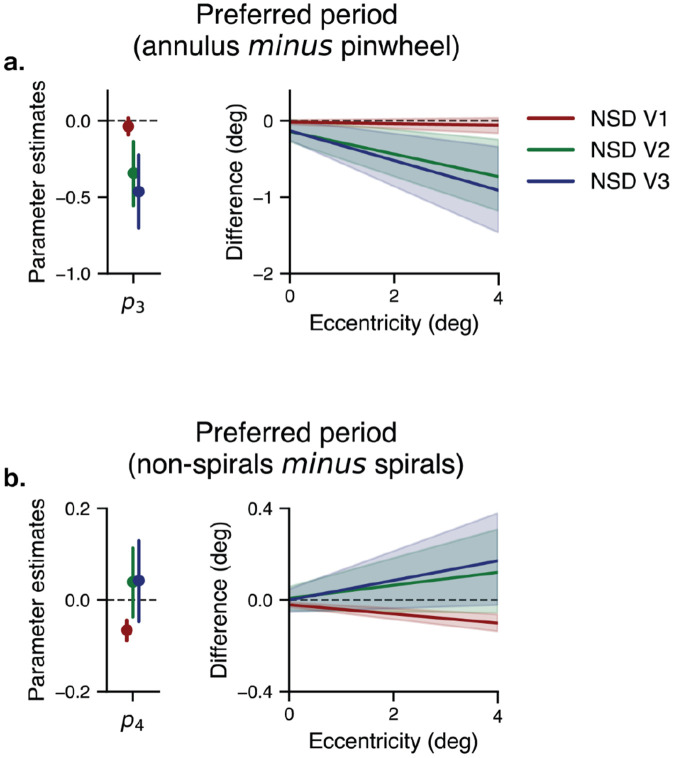
Relative orientation effect on preferred period of V2 and V3 in NSD (“Extension”), with NSD V1 from Figure 10 replotted. For all the panels a and b, the left column shows the estimated parameter values, with the dots and the error bars representing precision-weighted averages and 68% bootstrapped confidence intervals across subjects. The right column shows the difference in preferred period when local orientation is at 90° and retinotopic angle is at 180°. The difference was calculated between the two orientations on which each p parameter depends. a) The difference in preferred period between circular (“annulus”) and radial (“pinwheel”) orientations. b) The difference in preferred period between non-spiral (“annulus” and “pinwheel”) and spiral (“forward spiral” and “reverse spiral”) orientations.

As described in section 3.2.5, the two parameters for relative orientation, $p_{3}$ and $p_{4}$ were not consistent between Broderick et al and NSD for V1. Here we see that the parameters also show no systematic effects across V1, V2, and V3 in NSD. In NSD V1, $p_{3}$ is about 0 (no difference in preferred period for radial vs circular patterns) and $p_{4}$ is negative (higher preferred period for spirals than non-spirals). In contrast, the V2 and V3 estimates are negative for $p_{3}$ and about 0 for $p_{4}$, but there is a lot of variability across subjects in the V2/V3 estimates of $p_{3}$ and $p_{4}$, making it difficult to determine whether the differences from V1 are meaningful (Figure 16).

#### 3.3.6. Extension summary

**Figure 17.**
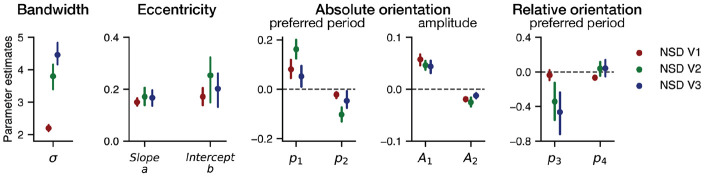
2D model fits for all the nine parameters of NSD V1, V2, V3.

First, we observed that in NSD, there were reliable responses to our stimuli in V2 and V3, with clear spatial frequency tuning. This enabled us to quantify the effect of eccentricity on peak spatial frequency tuning, which was similar to the effects in V1. The most salient difference between the extrastriate areas and V1 was the increase in bandwidth. Moreover, the model fits show similar effects of absolute orientation in the extrastriate maps in V1– higher preferred period and higher amplitude for vertical than horizontal stimuli ($p_{1}$, $A_{1}$) and for oblique than cardinal stimuli ($p_{2}$, $A_{2}$). The effects of relative orientation were less systematic ($p_{3}$, $p_{4}$). A summary of the 9 parameters for all three visual areas in NSD is plotted in Figure 17.

## 4. Discussion

We measured and modeled spatial frequency tuning in human visual cortex using data from the NSD. We implemented the same model of V1 spatial frequency tuning as described in Broderick et al. (2022), and compared our parameters to theirs for V1. The bandwidth, effects of eccentricity, and effects of absolute orientation (cardinal vs. oblique; horizontal vs. vertical) were similar in the two datasets, whereas effects of relative orientation (circular vs. radial) differed. We then applied the model to V2 and V3 data from NSD, and compared the model parameters to those for V1. The most salient difference between the extrastriate maps and V1 was an increase in bandwidth, doubling from V1 to V3.

### 4.1. Computational Reproducibility, Empirical Reproducibility, and Generalization

Reproducibility has become a topic of intense interest in psychology (Brandt et al., 2014; Klein et al., 2014; Open Science Collaboration, 2015), neuroscience (Bossier et al., 2020; Gilmore, Diaz, Wyble, & Yarkoni, 2017), and biomedical research (Mullane et al. 2018; Ioannidis et al. 2001), with some claims that the majority of research findings do not replicate (Ioannidis 2005). Here, we discuss replication from multiple perspectives.

First, an important question for computational research is whether the same results are obtained if one re-analyzes the same data, i.e., *computational reproducibility*. While this might appear to be a low bar, leaders in computational research have in fact noted that “error is ubiquitous in scientific computing, and one needs to work very diligently and energetically to eliminate it” (Donoho, 2010). Or, more colorfully put by Einstein, “Death alone can save one from making blunders” (Einstein & Born, 2004). Indeed, many papers with computational models cannot be easily replicated due to the lack of publicly available code and/or data and because re-analysis of the same data with new code implementation often leads to different results (Botvinik-Nezer et al., 2020; Li et al., 2024; Stodden et al., 2016). The challenge of computational reproducibility was lowered in our case as two of the authors of this manuscript were also authors of the original paper. Even so, completely new code was implemented here by the first author of this paper who was not involved in the prior paper, and this code was applied de novo to the publicly shared data from the original paper (http://hdl.handle.net/2451/63344). We note that the process of re-analyzing the original data with new code was complex, effortful, and instructive, and even led to the discovery of a minor error in the original paper (https://doi.org/10.1167/jov.24.13.8). In fact, we discovered two errors which mostly canceled each other, and hence did not change the estimated parameter values, though it did result in an incorrect description of some of the stimuli.

*Empirical reproducibility* refers to whether one obtains the same results from performing an identical (or nearly identical) study. *Generalization* refers to whether the same results hold when some important aspect of the study is varied. In practice, there is not a sharp divide between the two since there are inevitably some differences between a replication study and the original study. The analysis here serves as a test somewhere between empirical reproducibility and generalization: the 2D model used to analyze the data was identical to that used by Broderick et al., and the scaled grating stimuli were highly similar, but there were several critical differences, including magnetic field strength, fMRI acquisition and processing, experimental design, stimulus size (Table 1). Many of the parameter estimates from Broderick et al were similar in the NSD dataset, including bandwidth, effects of eccentricity, and effects of absolute orientation, indicating good reproducibility and generalization across experimental conditions. The two parameters pertaining to relative orientation (annuli vs pinwheels, and spirals vs non-spirals) did not replicate. This might be due to differences in the experiments, especially the eccentricity range of the stimuli, but we cannot definitively rule out the possibility of a simple replication failure, meaning the possibility that the original effects were spurious. The reproducibility of the bandwidth and eccentricity effects is important: prior fMRI studies on spatial frequency tuning in V1 have reported peak spatial frequency ranges spanning about 5-fold (Broderick et al. 2022; Aghajari et al. 2020; D'Souza et al. 2016; Farivar et al. 2017; Henriksson et al. 2008; Sasaki et al. 2001; Hess et al. 2009; Kay 2011). Now, including this study, three recent papers have converged to close agreement on peak spatial frequency tuning as a function of eccentricity in V1 (Aghajari et al., 2020; Broderick et al., 2022).

### 4.2. Increasing bandwidth along visual hierarchy

The similarity of our V1 model parameters to those of Broderick et al provides some assurance that our V1 model is reasonable, and justifies our comparison of the V1 results to those of extrastriate cortex in NSD. The most salient difference between V1 and extrastriate spatial frequency tuning was the increase in bandwidth from V1 to V2 to V3. This increase parallels the pRF size increases from V1 to V2 to V3 (Dumoulin & Wandell, 2008) and the size increases in single neuron RFs from V1 to V2 to V3 (Burkhalter & Van Essen, 1986; Felleman & Van Essen, 1987; Gattass, Gross, & Sandell, 1981; Gattass, Sousa, & Rosa, 1987). The presumption is that increasingly large receptive fields reflect the pooling of outputs of many neurons from previous areas (Van Essen & Maunsell, 1983). For example, Harvey and Dumoulin (Harvey & Dumoulin, 2011) accurately predict V2 pRF size by assuming that each location in V2 pools over about 3 mm of V1 (standard deviation of a 2D Gaussian pooling function). The same pooling of many V1 neurons by each V2 neuron might be expected to widen the spatial frequency tuning in V2, similar to widening the spatial tuning (i.e., the receptive field size). However, this explanation does not provide a quantitative match: We find that given our estimate of spatial frequency tuning functions across the V1 map, simply blurring the V1 representation with a 3 mm kernel results in only a tiny increase in bandwidth for V2 (less than 1%), much smaller than the 70% increase in bandwidth we observed. Hence, additional factors likely contribute to the large increase in bandwidth from V1 to V2.

Why would a simple blur function across the cortical surface accurately predict increases in pRF size but not increases in spatial frequency bandwidth? We believe several factors are at play. First, we note that preferred spatial frequency changes relatively slowly across the cortical surface compared to the change in position tuning. Hence, blurring alone is not expected to yield large increases in spatial frequency bandwidth. Second, we suggest that the greater bandwidth in V2 might arise from specific nonlinear neural computations. For example, V2 neurons may exhibit compressive non-linearities in their pooling over responses of V1 neurons. This possibility is supported by the observations of increasing non-linearities in spatial tuning (Kay, Winawer, Mezer, & Wandell, 2013), temporal tuning (Zhou, Benson, Kay, & Winawer, 2018), and contrast sensitivity (Kay, Winawer, Rokem, Mezer, & Wandell, 2013). For example, consider a high spatial frequency pattern that only weakly drives V1 neurons in a patch of cortex. V2 neurons that pool the responses of V1 neurons nonlinearly might amplify the weak response, effectively increasing the V2 bandwidth. A possible alternative explanation is that there are a relatively small number of V1 neurons tuned to very low frequencies or very high frequencies, and that V2 oversamples these outputs. We cannot distinguish the two possibilities with the current data and analyses.

Our finding of increasing bandwidth from V1 to V2 to V3 differs from Aghajari et al. (2020), who found similar bandwidths in the three areas. The estimates in V1 are similar, diverging much more in V2 and V3. For example, at 5 deg eccentricity, our estimates are 5.2, 9, and 10.6 octaves for V1, V2 and V3, whereas for Aghajari et al. (2020), the corresponding estimates are all about 4.5 octaves (full-width at half max). The difference could arise from stimulus differences, as we used scaled gratings and they used band-pass noise. Neural encoding in extrastriate visual areas is more sensitive to higher order image statistics, including second order contrast (Freeman, Ziemba, Heeger, Simoncelli, & Movshon, 2013; J. Anthony Movshon & Simoncelli, 2014; Okazawa, Tajima, & Komatsu, 2017), and as a result, specific quantitative measurements of spatial frequency tuning may exhibit dependence on other properties of the stimulus not considered in the data analysis. Additionally, our parameter estimates came from fitting a single model to a whole visual area (2D-model) or computing tuning curves to the average across many voxels (1D model), whereas Aghajari fit models to each voxel independently. Because of the nonlinear relationship between model parameters and response amplitudes, averaging the model parameters across voxels can give different results from fitting the model parameter to the average across voxels.

### 4.3. Opposing effects on amplitude and preferred spatial frequency

A curious finding is that stimulus orientations that have higher preferred spatial frequency elicit lower amplitude responses. This was evident in the $p_{1}$ and $A_{1}$ parameters (vertical > horizontal) and the $p_{2}$ and $A_{2}$ parameters (oblique > cardinal). In other words, for horizontal orientations compared to vertical, the preferred spatial frequency is higher and the amplitude is lower. And similarly, for cardinal orientations compared to oblique, the preferred spatial frequency is higher and the amplitude is lower. These patterns were consistent, in that they were found in both Broderick et al. and NSD V1, and in NSD V2 and V3. One might expect that tuning to higher frequencies would support better visual acuity; for example the foveal representations in V1 to V3 are tuned to higher frequencies, and foveal acuity is much higher than peripheral acuity. Similarly, both grating acuity (Berkley et al., 1975) and vernier acuity (Corwin, Moskowitz-Cook, & Green, 1977; Leibowitz, 1955) are higher for cardinal than oblique orientations, paralleling our finding that spatial frequency tuning is higher for cardinal stimuli. There is not, however, clear evidence that acuity is higher for horizontal than vertical orientations (Berkley et al., 1975; Campbell et al., 1966; Long & Tuck, 1991). Thus the link between the cortical tuning measured by fMRI and behavior is not straightforward. Moreover, higher BOLD amplitude for oblique than cardinal orientation is not straightforward to link to poorer performance on most tasks with oblique orientations than cardinal orientations, as stimuli that elicit higher amplitude neural responses are often associated with better behavioral performance, at least on detection tasks (Ress, Backus, & Heeger, 2000).

More generally, there are many challenges in linking behavioral performance to neural data. One is that the neural measures, as in this paper, are made with high contrast stimuli to elicit large responses, whereas behavioral measures are often made at low contrast to measure thresholds. Second, without designing the behavioral and neural studies together, there are likely to be differences in stimuli (duration, eccentricity, spatial extent, and so on), subject populations, and other methods. And third, perhaps most importantly, a neural circuit model of the task is needed in order to know how the neural measure should limit performance (Kay, Bonnen, Denison, Arcaro, & Barack, 2023). Such models are difficult to implement for most kinds of experiments (Morgan, Melmoth, & Solomon, 2013). Although we do not offer linking models in this paper, we hope that the data we report, including the measures of variability between subjects and replicability across subjects, can serve as useful constraints in the development of such models.

## 5. Conclusions

Three reports, including this one, now show quantitative agreement on the spatial frequency tuning in human V1 as a function of eccentricity (Broderick et al. 2022; Aghajari et al. 2020; D'Souza et al. 2016; Farivar et al. 2017; Henriksson et al. 2008; Sasaki et al. 2001; Hess et al. 2009; Kay 2011), despite differences in stimuli, analysis methods, and scanners. This is an advance over where the field stood just a few years earlier, where the range of reported values for peak spatial frequency tuning in V1 varied several-fold across studies. Much of the advance is likely due to a combination of stimulus selection, spanning a wide range of spatial frequencies, and improvements in analysis methods. The increased precision in the characterization of spatial frequency tuning has further enabled replicable measures of more subtle effects. In particular, the effect of absolute stimulus orientation on spatial frequency tuning also replicated across studies. Finally, the most novel observation reported here is that the bandwidth of spatial frequency tuning increased substantially from V1 to V2 and V3, suggesting a parallel between position tuning and spatial frequency tuning: both receptive field size and spatial frequency bandwidth increase along the cortical visual hierarchy.

## Supplementary Material

Supplement 1

## Figures and Tables

**Table 1.** Different experimental implementation between the two datasets. Left column shows details of experimental implementations in Broderick et al. (2022), whereas the right column shows those in the NSD gratings dataset.

	Broderick et al. (2022) dataset	NSD gratings dataset
Number of subjects	12	8
Stimulus diameter (deg)	24.4 (circular)	8.4 (square)
Number of base frequencies	10	6
Number of orientations	4	4
Number of trials per condition	12	8
Number of images per trial	8	1
Task	fixation-related	fixation-related or image-related
