# Supplementary material for: Spatial Frequency Maps in Human Visual Cortex: A Replication and Extension
Source: bioRxiv. 2025 Feb 5:2025.01.21.634150. Originally published 2025 Jan 21. Preprint. [Version 2] doi: 10.1101/2025.01.21.634150 (PMC11785079; doi:10.1101/2025.01.21.634150)
Supplement: Supplement 1 [file media-1.pdf]

Supplementary Information

Table 1. Frequency information of experimental stimuli.

**Stimulus Class**	ω_r	ω_a	ω	$\omega_{l=2º}$
Pinwheels	0	6	6	3
	0	11	11	5.5
	0	20	20	10
	0	37	37	18.5
	0	69	69	34.5
	0	128	128	64
Annuli	6	0	6	3
	11	0	11	5.5
	20	0	20	10
	37	0	37	18.5
	69	0	69	34.5
	128	0	128	64
Forward spirals	4	4	≈5.66	≈2.83
	7	7	≈9.90	≈4.95
	14	14	≈19.80	≈9.90
	26	26	≈36.76	≈18.38
	49	49	≈69.29	≈34.64
	91	91	≈128.67	≈64.34
Reverse spirals	4	-4	≈5.66	≈2.83
	7	-7	≈9.90	≈4.95
	14	-14	≈19.80	≈9.90
	26	-26	≈36.76	≈18.38
	49	-49	≈69.29	≈34.64
	91	-91	≈128.67	≈64.34
Mixtures	14	34	≈36.78	≈18.39
	34	14	≈36.78	≈18.39
	34	-14	≈36.78	≈18.39
	14	-34	≈36.78	≈18.39
